# Detection frequencies and viral load distribution of parvovirus B19 DNA in blood and plasma donations in England

**DOI:** 10.1111/tme.12893

**Published:** 2022-06-25

**Authors:** Sarah Williams, Jeremy Ratcliff, Dung Nguyen, Peter Simmonds, Heli Harvala, International Survey Group

**Affiliations:** ^1^ Nuffield Department of Medicine, Peter Medawar Building for Pathogen Research University of Oxford Oxford UK; ^2^ Microbiology Services, NHS Blood and Transplant UK

## Abstract

**Background and Objectives:**

Infections with human parvovirus B19 (B19V) are transmissible by blood components and plasma‐derived medicines. The European Pharmacopoeia regulates maximum levels of virus allowed in manufacturers' plasma pools. To evaluate contamination risk prior to re‐introduction of UK‐sourced plasma for manufacturing, we investigated viraemia frequencies of B19V in plasma samples collected from blood donors before and during COVID‐enforced lockdown.

**Materials and Methods:**

Quantitative PCR for B19V DNA was used to screen pools of 96 anonymised plasma samples collected in England from 2017 (*n* = 29 505), 2020 (*n* = 3360) and 2021 (*n* = 43 200). Selected positive pools were resolved into individual samples. Data on donor notifications and related lookback investigations were collected from European countries by on‐line survey in 2020.

**Results:**

Screening of 76 065 donations identified 80 B19V‐positive pools. While most positive samples had low viral loads (<10^5^ IU ml^−1^), primarily from 2017 (77/29 505; 0.3%), two contained high levels of B19V DNA (1.3 × 10^8^ and 6.3 × 10^6^ IU ml^−1^), both likely to contaminate a final manufacturer's pool and lead to discard. The incidence of B19V infection during lockdown was reduced (1/3360 in 2020; 0/43 200 in 2021). Genomic analysis of positive pools resolved to single samples identified B19V genotype 1 in all nine samples. Seroprevalence of anti‐B19V IgG antibodies was 75% (143/192). A survey of B19V screening practices in Europe demonstrated considerable variability. Two blood establishments informed infected blood donors of positive B19V results.

**Conclusion:**

Information on seroprevalence, incidence and viral loads of B19V viraemia is contributory the evaluation of alternative operational screening strategies for plasma testing.

## 1 INTRODUCTION

Infections with human parvovirus B19 (B19V) are associated with intense viraemia and blood donations collected during the acute phase have been shown to transmit infections to recipients of red cells, platelets and plasma‐derived blood products.^1^ B19V is a small non‐enveloped DNA virus, with three known genotypes that infect humans.^2^ In immunocompetent individuals, B19V infections are largely asymptomatic although by targeting of erythroid progenitor cells in the bone marrow, B19V creates a temporary reduction in reticulocytes as well as in circulating lymphocytes, neutrophils and platelets.^3^ More severe infection outcomes such as prolonged anaemia or transient aplastic crisis may therefore occur in those with pre‐existing haematological diseases, such as sickle cell anaemia^4^ or autoimmune haemolytic anaemia.^5^ Infections acquired during early pregnancy (<18 weeks) may lead to hydrops fetalis.^6^

The intense viraemia that occurs during acute infections has led to documented instances of transfusion transmitted B19V infections, first described in the 1990s.^1^ B19V may be transmitted by all blood components (red cells, platelets, fresh frozen plasma, cryoprecipitate) and also through pooled plasma products (reviewed in reference 7). The latter have a high probability of infectivity, given the large pool sizes and individual plasma donations with extremely high viral loads associated with primary infections up to 10^14^ DNA copies /ml^8^, ^9^;. These may contaminate an entire manufacturing pool. B19V infectivity is also relatively resistant to inactivation by heat, detergents or commercial pathogen inactivation methods such as Intercept (Cerus) during the fractionation process used to manufacture immunoglobulins and other products from plasma.^10^, ^11^ NAT screening to eliminate highly viraemic donations has therefore been widely adopted to reduce the possible risk of B19V transmission by plasma‐derived products.^12^, ^13^, ^14^ The European Pharmacopoeia accordingly specifies a requirement that plasma pools used for manufacturing, typically comprising between 6000 and 24 000 individual donations, should contain B19V DNA loads of less than 10 000 international units (IU)/ml.^15^, ^16^ This cut‐off was determined based on calculations of residual infectivity following virus inactivation. It is mandatory to discard all final manufacturers' plasma pools exceeding these levels of B19V DNA.

Development of effective strategy for B19V screening of plasma destined for fractionation in the UK has become a priority. The use of UK‐sourced plasma was discontinued in 1998 in response to concerns over the spread of variant Creutzfeldt Jakob Disease (vCJD). However, the absence of diagnosed cases of vCJD cases in the UK since 2016 after mandatory changes introduced in animal industry led to a comprehensive review of the evidence of the safety of UK plasma for the manufacture of immunoglobulins over 20 years later.^17^ It concluded that it would be safe to use UK‐sourced plasma providing robust safety standards and other risk mitigation measures remained in place.

However, re‐starting plasma product manufacturing from UK‐sourced plasma requires consideration of how plasma might be efficiently tested for B19V. A crucial decision is whether to implement testing of component plasma units to identify and exclude highly viraemic donations only (>10^6^ IU ml^−1^) that would contaminate final manufacturing pools over the regulatory threshold. To investigate this, we have evaluated a previously established RT‐PCR assay for high‐throughput quantitative detection of B19V DNA,^18^ and generated baseline data on the incidence of B19V viraemia and associated viral loads in blood donors in England between 2017 and 2021. B19V variants in positive samples were genomically characterised to determine infecting genotypes and any potential epidemiological linkage between infected donors.

The time points were selected to further investigate potential changes in infection frequencies of B19V during the COVID pandemic; elsewhere, there is evidence that incidences of clinically reported cases of both have declined substantially during the prolonged periods of lockdown designed to interrupt the transmission of SARS‐CoV‐2.^19^, ^20^ We have explored the operational implications of introducing B19V screening if it was to be introduced in England for donor follow‐up and potential lookback investigations.

## 2 METHODS

### 2.1 Quantitative B19V PCR

A previously described method for B19V DNA and HAV RNA detection by PCR was used.^18^ Analytical sensitivities of the PCRs were determined for all three B19V genotypes by assaying serial dilutions of each WHO International Standard in 50 ng μL^−1^ DNA carrier (Table S1; Supporting Information). Probit analysis was used to determine the 95% limit of detection (LOD) for the assays using SPSS version 26.

International standards for the three B19V genotypes were obtained from the National Institute of Biological Standards and Control (NIBSC, London, UK; code 09/110).

### 2.2 B19V testing

A volume of 200 μm of pools comprising 96 plasma samples were initially screened in replicate following nucleic acid extraction using the Quick‐DNA/RNA Viral Kit (Zymo Research, Cambridge Bioscience, UK). One fifth of the eluate (representing 40 μl of original sample) was used in the PCR. Pools showing positive reactivity (Ct values <40) in one or both replicates were re‐tested. Pools showing positivity in 3/4 or 4/4 combined replicates were assigned as positive; those showing reactivity in 1/4 replicates were assigned as negative. Those positive in 2/4 replicates were retested in replicate in a third PCR. Those showing reactivity in 3/6 or 4/6 of replicates were assigned as positive. Those negative on the third PCR were scored as negative (2/6 reactive overall).

Selected pools showing a range of viral loads were split into their eight component minipools of 12 plasma samples and nucleic acid was extracted from these minipools. Upon identification of one or more positive minipools of 12, the 12 individual samples within each were identified with individual sample PCR.

### 2.3 B19V sequencing

B19V DNA from nine positive samples were amplified by nested primers using primers spanning the VP2 region and sequenced via sanger sequencing (Table S1, Supporting Information). Sequence data were read between positions 3876 and 4953 (positions numbered relative to the AY386330 reference sequence) and compared with available B19V complete genome sequences in this region (sequences listed in Table S2, Supporting Information). Phylogenetic analysis of B19V nucleotide sequences was performed by using the program MEGA6.^21^

### 2.4 B19V serology testing

Subsets of donor samples were assayed for B19V IgG (*n* = 192) and IgM (*n* = 16) antibodies using the Serion ELISA for parvovirus B19 IgG/IgM following the manufacturer's instructions (Wurzburg, Germany). Testing was extended to assay IgG and IgM antibodies in the 10 individual samples identified B19V DNA positive by screening. Samples were assigned as positive, indeterminate, or negative based on the manufacturer's criteria.

## 3 SUBJECTS STUDIED

### 3.1 Plasma samples and controls

Three groups of anonymised plasma samples were obtained from NHS Blood and Transplant (NHSBT):

- Twenty nine thousand five hundred and ninety two archive donations collected in September 2017 in England in the pre‐pandemic period.
- Three thousand three hundred and sixty samples from plasma donors in 2020 enrolled in the SARS‐CoV‐2 convalescent antibody programme.
- One thousand eight hundred residual NAT minipools each containing 24 samples (total 43 200 individual donations) collected betweenJanuary and February 2021 for HBV/HIV/HCV and HEV RNA screening by Roche Nucleic Acid Testing (NAT).

Although the sources of the 76 065 samples at the three points varied, this did not affect the representativeness of donors of blood and plasma over these periods as they were collected from a large number of geographically dispersed donor centres in England, and comparable in age ranges and gender.

### 3.2 Ethical statement

Signed consent was obtained from each donor at the time of donation. This included consent to NHSBT to use their data for the purposes of clinical audit to assess and improve our services as well as to increase our knowledge of the donor population.

### 3.3 Survey

The potential gain from the introduction of pre‐testing of donations before manufacturers' pooling was evaluated against current practice in Europe. In the absence of published data on testing policies as regards to screening methods, assay sensitivities, viral load thresholds and actions taken in the event of positive donations being detected, we performed a survey of current screening practice in European Blood Establishments in September 2020.

## 4 RESULTS

### 4.1 Sensitivity and reproducibility of the B19V PCR

The analytical sensitivity of the B19V DNA assay was determined using dilution series of the WHO International Standards (Table S3, Supporting Information). From these, the 95% LODs were calculated by Probit analysis for the three B19V genotypes (Table 1). They ranged from 29 IU ml^−1^ in VP2 for B19V genotype 2 to 365 IU ml^−1^ for B19V genotype 1 using NS1 primers (Table 2).

**TABLE 1:** Sensitivity and reproducibility of B19V PCR—lower limits of detection

Virus	Region	IU	IU mL^−1^ ^a^
*B19V*			
Genotype 1	NS1	14.6	365
	VP2	7.0	175
Genotype 2	NS1	1.3	38
	VP2	1.2	29
Genotype 3	NS1	4.2	105
	VP2	2.7	67

^a^ Based on extraction of 200 μl of sample, elution into 50 μl of which 10 μl was amplified by PCR.

**TABLE 2:** Sensitivity and reproducibility of B19V PCR—assay Ct value and inter‐sample variability

IU	N^a^	NS1^b^	VP2
5000	13	28.16 (0.23)	27.36 (0.48)
500	29	32.34 (1.23)	31.20 (0.97)
50	29	35.57 (1.79)	34.84 (1.89)
5	26	38.29 (1.14)	37.48 (1.12)

^a^ N: number of replicates tested.

^b^ Ct value: mean (± SD).

Multiple testing of the B19V genotype 1 standard produced Ct values with relatively low variability between values on replicate testing (Table 2). For example, a mean Ct value of 28.16 and standard deviation (SD) of ±0.23 was recorded for 5000 IU (1.25 × 10^5^ IU ml^−1^) dilution of the genotype 1 standard using the NS1 primers and 27.36 (SD ± 0.48) for VP2. Inter‐assay variability was comparable for other dilutions (Table 2).

### 4.2 Screening of blood donations

**FIGURE 1:**
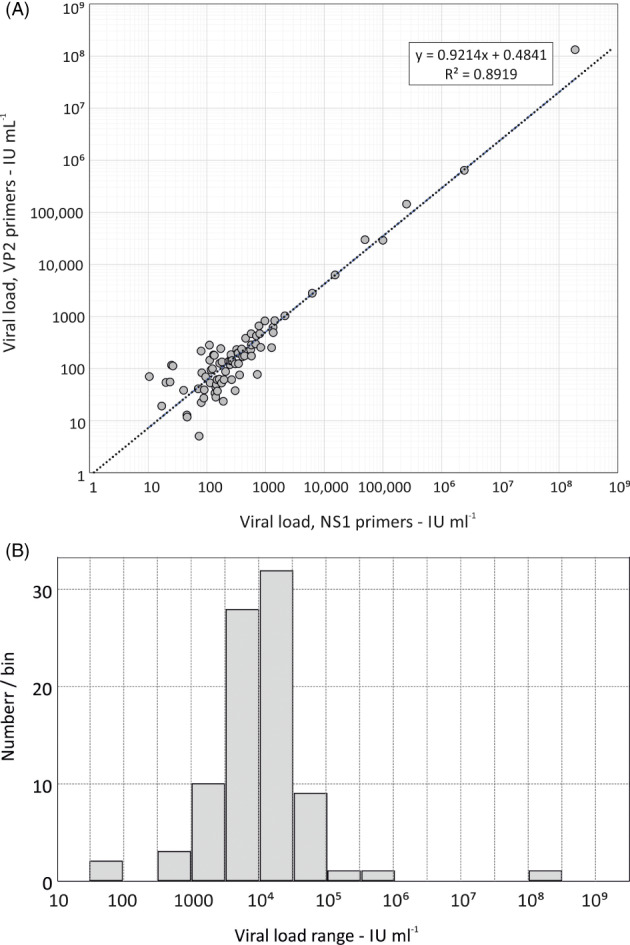
(A) Correlation between viral load quantitation in study samples in NS1 and VP2 B19V PCR assays. A regression line of log transformed viral loads in the two assays is shown as a dotted line; the formula for the regression line and correlation coefficient, *R* ^2^, are shown in the box. (B) Distribution of viral loads of study samples quantified by NS1 PCR. All viral loads of the B19V positive samples identified on screening were based on values calculated from individual testing or from pooled testing, expressed in IU ml^−1^ by reference to co‐tested calibrated run controls of B19V genotype 1

We screened 76 065 plasma samples in pools of 96 for B19V DNA; 29 505 from 2017, 3360 in 2020 and 43 200 in 2021. On initial screening of the 793 pools, a total of 80 positive pools were identified (Table 3; Table S4, Supporting Information). Viral loads obtained by NS1 and VP2 PCRs closely correlated (Figure 1A) and spanned a wide range from <100 to >10^8^ IU ml^−1^ (Figure 1B). Two samples containing high levels of B19V DNA (1.32 × 10^8^ IU ml^−1^ and 6.37 × 10^5^ IU mL^−1^ in the VP2‐based PCR) were identified from donations collected in 2017 (1:14752). Apart from a single PCR‐positive sample from 2020, all other B19 positive samples were also collected in 2017 (79:29505; 0.3%) (Table 3). The remaining sample with low‐level B19V DNA was identified in convalescent plasma donor in 2020 (1:3360; 0.03%).

**TABLE 3:** Detection frequencies of B19V DNA and HAV RNA in study samples—viraemia frequencies

Virus	Period	Donations	PCR Positive	Frequency	Total > 10^5^ IU m^−1^	Frequency	Sero‐positivity
B19V	All	76 065	80	0.11%	2	0.003%	–
	2017	29 505	79	0.27%	2	0.007%	143/192
	2020	3360	1	0.030%	0	–	–
	2021	43 200	0	–	0	–	–

A selection of positive pools of 96 samples over a range of viral loads were split to individual samples of minipools and tested (Table 4); from these, 6 out of 8 minipools yielded single positive sample and the remaining two minipools yielded two. Viral loads in the samples were comparable to those calculated from pooled testing taking the dilution factor into account. Comparison of VP2 sequences from nine of the resolved single donation samples was performed to investigate potential epidemiological linkage between infected donors and rule out contamination as a cause of the observed positive test results. VP2 sequences from nine of the resolved single donation samples were all of genotype 1, but with little apparent linkage between strains infecting different donors (Figure S1, Supporting Information).

**TABLE 4:** Detection frequencies of B19V DNA and HAV RNA in study samples—testing of component donations

Pool ID	Pools of 12^a^	Single^a^	Individual donation	NS1 VL^b^	VP2 VL^b^	B19V IgG^c^	B19V IgM^c^
A001	1	1	A001/A10	9.28 × 10^2^	2.07 × 10^2^	Pos (2.1)	Pos (3.2)
B081	1	1	B081/C3	1.25 × 10^4^	2.97 × 10^4^	Pos (2.8)	Neg (0.4)
B110	1	1	B110/C12	1.59 × 10^c^	2.79 × 10^c^	Pos (2.7)	Neg (0.5)
B112	1	1	B112/C2	nd	3.86 × 10^2^	Pos (2.7)	Neg (0.2)
B122	1	2	B122/F2	1.96 × 10^b^	6.60 × 10^b^	Pos (2.6)	Neg (0.3)
			B122/F11	2.52 × 10^4^	2.90 × 10^4^	Pos (2.6)	Pos (1.8)
B219	1	1	B219/D11	4.77 × 10^7^	1.32 × 10^8^	Neg (0.1)	Neg (0.2)
B256	1	2	B256/G5	6.39 × 10^4^	1.44 × 10^5^	Pos (2.6)	Pos (3.2)
			B256/G12	3.89 × 10^3^	6.27 × 10^3^	Pos (2.6)	Neg (0.6)
B280	1	1	B280/E5	6.15 × 10^5^	6.37 × 10^5^	Pos (1.5)	Pos (3.4)

^a^ Number of positive sub‐pools on splitting.

^b^ Units in IU ml^−1^; shaded by viral loads.

^c^ Net ODs after subtraction of substrate blank; shaded by reactivity; OD ranges for result assignments: IgG assay: positive (grey and black filled cells): >0.42; indeterminate: <0.42, >0.29; Negative: <0.29 (unfilled cells); IgM assay: positive: >0.41 (grey and black filled cells); indeterminate: <0.41, >0.29 (unfilled cells); Negative: <0.29 (unfilled cells).

High rates of past exposure to B19V were apparent on testing a selection of individual samples from 2017 for B19V IgG antibodies by ELISA (Tables 3 and 4). The overall seropositivity was 74.5% (143/192 samples positive), with a further three showing equivocal reactivity. Individual positive samples identified through splitting pools were assayed for IgG and IgM anti‐B19V antibodies by ELISA (Table 4). All seven samples with viral loads <10^5^ IU ml^−1^ were seropositive for IgG and either negative or weakly reactive for IgM (2 negative, 2 indeterminate, 3 positive). This contrasts with 15/16 IgM negative, 1/16 IgM indeterminate in 16 randomly selected PCR negative donor samples (data not shown). Contrastingly, the sample with the highest viral load (1.3 × 10^8^ IU ml^−1^) was seronegative for IgG and IgM, while two samples with viral loads >10^5^ IU ml^−1^ were IgG positive and strongly IgM positive, in all three cases consistent with proximity to seroconversion.

### 4.3 Survey results

A total of 12 blood establishments providing plasma for fractionation, all members of the European Blood Alliance (EBA), responded to our donor survey. Testing methods (i.e. from individual [ID] NAT to pooling of up to 512 donations, commercial platforms or in‐house methods) and policies for the initiation of clinical follow up of viraemic donors and blood component recipients varied greatly between centres (Table 5). Only one blood establishment provided plasma for fractionation without additional B19V testing. The fractionator performed B19V testing for five blood establishments in minipools, but without results being provided back to the blood establishment or blood donors in four cases. One blood establishment receiving results informed their donors of B19V results. Of the remaining six establishments, five undertake B19V testing of their own donations and one sends their samples to another country. A range of commercial (Roche, Grifols) and in‐house assays were used. Pool sizes for screening were typically 96. Of these six, only one country informs the donors of their B19V result. None of the respondents of the survey indicated that their blood establishments informed recipients of transfused blood components of their exposure to B19V.

**TABLE 5:** Survey results of B19V and HAV screening practice in Europe

Country	Estonia	Belgium Croix‐Rouge	Belgium Rode Kruis	Portugal	Italy	Switzerland	Finland	France	Germany	Austria	Netherlands	Slovenia
Plasma provided for fractionation?	Yes	Yes	Yes	Yes	Yes	Yes	Yes	Yes	Yes	Yes	Yes	Yes
Is plasma tested for B19V?	Yes	Yes	Yes	Yes	Yes	Yes	Yes	Yes	Yes	Yes	Yes	No
Who does B19V testing?	Fractionator	Fractionator	Fractionator	Fractionator	Fractionator	Referring Laboratories	In‐house	In‐house	In‐house	Other	In‐house	
What assay is used for B19V?	Not known	Not known	Not known	Not known	Not known	Commercial	Grifols	Grifols	Roche DPX		In‐house	
Confirmatory testing for B19V?						No	No	No	No	No	No	
Pool size for HBV/HCV/HIV NAT?	ID	8	6	ID	ID	ID	ID	ID	96	96	6	ID
Pool size used for B19V testing	Not known	Not known	Not known	96	<512	ID NAT	16	96	96		480/96	
Do you inform donors of B19V result?	No^a^	No^a^	No^a^	Yes	No	Yes (letter)	No	No	No		No	
How long you exclude donor with B19?							None	None	2 months		2 weeks^b^	

^a^ We do not receive parvovirus B19 results back from fractionator.

^b^ Only if reported symptoms.

## 5 DISCUSSION

Detection and quantitation of viraemia by PCR in blood donors provides one metric of the incidence of B19V infections in the survey population. The PCR assay for B19V DNA detection evaluated here showed a robust performance with the sensitivities and reproducibility as expected.^18^ The information obtained in the study is vital for the rational planning of the introduction of B19V screening of UK‐sourced plasma for manufacturing of medicines. In particular, knowledge of the current and likely future frequencies and viral loads of B19V viraemia are required to guide the choice of effective pool sizes for screening to ensure that final manufacturing pools are not contaminated with B19V leading to their discard.

The current study demonstrated a frequency of high‐level B19V viraemia at 1:14 752 in donations collected in 2017 that was comparable to those reported elsewhere in similar screening formats.^22^, ^23^, ^24^ For example, Kooistra et al.^22^ reported viral loads of 10^10^ IU ml^−1^ in approximately 1:30 000 donations, >10^9^ IU ml^−1^ in 1:23 600 donations and > 10^6^ IU ml^−1^ in 1:16000 donations in an investigation of 6.5 million blood donations in the Netherlands between 2003 and 2009. B19V infections typically show a 3–4‐year incidence cycle^23^, ^25^ with highest rates of B19V infection in 2013 and a lower peak in 2017.^25^ However, rates of B19V DNA detection varied between countries, with Dutch blood donors showing more frequent peaks of high level viraemia in 2013, 2015, 2017 and 2019,^24^ while B19V‐associated cases of erythema infectiosum in Belarus peaked in only in 2006 and 2015/2016.^26^ In B19V epidemic years more than 1 per 5000 donations have been shown to be viraemic, while only low levels of B19V DNA is evident in other times.^23^, ^24^, ^25^ No B19V DNA was detected in blood donations collected in 2021 in this study and only one low‐level positive in 2020. This virtual disappearance of B19V viraemia was much more marked than previously reported changes associated with the 2 or 4 yearly incidence cycle of B19V infections. As previously proposed,^24^ implementation of infection control measures to prevent respiratory virus transmission during the COVID‐19 pandemic may have had a major effect on B19V transmission too during periods of lockdown.

There was considerable variability of testing strategies for B19V in blood donations destined for fractionation by developed nations; for example NAT on minipools of varying sizes is employed by Germany and Austria, Belgium and the USA.^27^ Furthermore, there are examples of alternative testing strategies to pooled NAT; for example, the Netherlands perform IgG testing on individual samples and thus deems plasma safe to use if B19V IgG positive for at least 6 months.^22^ Japan performs a haemagglutination assay on individual donations for B19V antigen, with the possible imminent implementation of chemiluminescent enzyme immunoassay on pooled plasma.^28^ In contrast, many developed countries do not currently test individual blood donations for B19V DNA, including China, Korea and Australia,^29^ where they consider the risk of transfusion‐transmitted B19V tolerable, as most transfusion‐transmissions of B19V only manifest with mild or no clinical symptoms, and can be readily managed.

Of particular relevance for plasma product manufacturing and the regulations concerning acceptable viral loads in manufacturing pools, we report detection of two donors with high B19V viral loads measured by the VP2 PCR of 1.32 × 10^8^ and 6.37 × 10^5^ IU ml^−1^. Assuming that manufacturers pool size is at least 6000 donations, B19V screening requires donations with B19V loads over 10^8^ IU ml^−1^ to be excluded to avoid contamination of manufacturing pools above the 10^4^ IU ml^−1^ threshold specified by the European Pharmacopoeia. In practice, we recommend that B19V screening of plasma prior to supply to manufacturers should identify donations containing B19V DNA loads above 10^6^ IU ml^−1^. This translates to a potential annual rate of around 68 from 2 million donations collected per year in England. This cut‐off would provide a 2‐log safety margin for screening and hence be capable of reliably detecting such donations while accommodating a parallel requirement not to detect low level of B19V viraemia seen in the much larger number of donations (0.3% in 2017), as their inclusion in manufacturing pools poses no transmission risk.^30^, ^31^ B19V DNA can indeed remain detectable in the blood of immunocompetent individuals at lower levels (<10^5^ IU ml^−1^) for months or even years after acute infection; based on one study up to 1% of blood donations contained low levels of B19V DNA^32^ and as low as 0.006% in another.^22^ B19V DNA is also known to remain detectable life‐long in various tissues of immunocompetent adults after infection. These low levels of B19V DNA detected 6 months after the acute infection are considered to be non‐infectious DNA remnants.^33^ Furthermore, low‐level viraemia usually coexists with parvovirus B19V IgG antibodies which will likely further neutralise any potential infectivity of the virus making inclusion of these units in manufacturing even less likely to lead to transmission. Since these donations contain neutralising B19V IgG antibodies^.^ Their removal from fractionation might actually be disadvantageous as it would diminish B19V antibody levels in plasma pools and other plasma‐derived products.

Based on our survey results, the proposed testing for B19V DNA in pools of 96 is compatible with plasma screening programmes established elsewhere in Europe. The Red Cross in Germany excluded donations with B19V DNA higher than 10^5^ IU ml^−1^ whereas donations containing 2000 IU ml^−1^ of B19V DNA were excluded in Finland. In the Netherlands, the cut‐off has been set to around 10^6^ IU ml^−1^. Irrespective of where such testing would be done (in‐house, external laboratory or fractionator) and in contrast to some blood establishments in Europe, NHSBT has taken a view that positive results should be reported back to the blood donors and where these results relate to donations of whole blood, appropriate lookback investigations of recipients of blood components should be carried out. Although B19V infections are mostly asymptomatic, they can have severe consequences for an individual's health. Identification of donors with HAV infections or high level B19V viraemia will allow follow‐up of potential vulnerable contacts, including immunocompromised individuals, pregnant women and those with underlying haematological disease. However, it is important to note that follow up of donors for mandatory markers also vary significantly across Europe, and by following up donors with high‐level positive results, we generally aim to align ourselves to countries with similar epidemiological and public health surveillance systems.

In conclusion, our study provides the first systematic investigation of B19V viraemia frequencies and viral loads in English blood donors, and of the seropositivity for B19V that indicates the proportion susceptible to infection. These are relevant data that will help guide policy for operational screening and strategies for avoiding contaminated manufacturing for medicines pools. The ability to resolve positive pools to the level of individual positive samples enables notification and follow‐up of both the infected donors and recipients of blood components produced from the donation. However, the likely retrospective nature of B19V screening may mean that results cannot be provided in time to prevent transfusion of viraemic units. Information on donor infection frequencies nevertheless could further contribute to public health surveillance.

## AUTHOR CONTRIBUTIONS

HH conceived of the study and obtained funding and agreement from NHSBT for the project. SW, JR and DN performed the laboratroy work and analysis of the results. SW wrote the Methods section and part of the Results section. PS and HH performed further data analysis and co‐wrote and finalised the manuscript.

## CONFLICT OF INTEREST

The authors have no competing interests.

## Supporting information


**TABLE S1**. SEQUENCES OF PRIMERS USE FOR NUCLEOTIDE SEQUENCING
**TABLE S2** ACCESSION NUMBERS OF (NEAR COMPLETE) B19V GENOME SEQUENCES
**TABLE S3** FREQUENCY OF POSITIVES ON SERIAL DILUTION OF B19V INTERNATIONAL STANDARD
**TABLE S4** ALL POSITIVE RESULTS (MEAN IU/ML)
**FIGURE S1** PHYLOGENETIC TREE OF AMPLIFIED SEQUENCES IN THE B19V VP2 REGION FROM STUDY SAMPLES AND GENOTYPE 1–3 B19V VARIANTS
