# Supplementary material for: Detection frequencies and viral load distribution of parvovirus B19 DNA in blood and plasma donations in England
Source: Transfus Med. 2022 Jun 25;32(5):402–9. doi: 10.1111/tme.12893 (PMC9796365; doi:10.1111/tme.12893)
Supplement: Supplementary file 1 — TABLE S1. SEQUENCES OF PRIMERS USE FOR NUCLEOTIDE SEQUENCING TABLE S2 ACCESSION NUMBERS OF (NEAR COMPLETE) B19V GENOME SEQUENCES TABLE S3 FREQUENCY OF POSITIVES ON SERIAL DILUTION OF B19V INTERNATIONAL STANDARD TABLE S4 ALL POSITIVE RESULTS (MEAN IU/ML) FIGURE S1 PHYLOGENETIC TREE OF AMPLIFIED SEQUENCES IN THE B19V VP2 REGION FROM STUDY SAMPLES AND GENOTYPE 1–3 B19V VARIANTS [file TME-32-402-s001.docx]

**SUPPLEMENTARY METHODS AND DATA.**

NESTED PCR AND SANGER SEQUENCING

Samples positive by qPCR were amplified via a two-step nested PCR. For the 1^st^ round amplification, 5 μL of extracted DNA was added to a master mix containing 5 μL of 5X GoTaq green reaction buffer, 2 μL of 2.5 mM dNTPs, 1.25 μL of 10 μM 1^st^ round sense primer, 1.25 μL of 10 μM 1^st^ round antisense primer, 10.375 μL of ultrapure water, and 0.125 μL of GoTaq G2 DNA Polymerase. For the 2^nd^ round amplification, 1 μL of 1^st^ round product was added to a master mix containing 5 μL of 5X GoTaq colorless reaction buffer, 2 μL of 2.5 mM dNTPs, 1.25 μL of 10 μM 2^nd^ round sense primer, 1.25 μL of 10 μM 2^nd^ round antisense primer, 14.375 μL of ultrapure water, and 0.125 μL of GoTaq G2 DNA Polymerase. For both rounds of amplification, the thermal cycler settings were: 94C for 3m, 30 cycles of 94C for 30s, 50C for 30s, and 72C for 1m15s, and a final extension of 72C for 5 min. Successful generation of second round amplicons was confirmed by gel electrophoresis. Nine amplicons underwent sanger sequencing by Source Bioscience (Cambridge, UK) using the 2^nd^ round antisense primer. Oligonucleotides were synthesized by IDT Technologies and are listed in Table S1.

TABLE S1

SEQUENCES OF PRIMERS USE FOR NUCLEOTIDE SEQUENCING

| **Primer** | **Position^1^** | **Orientation** | **Sequence** |
| --- | --- | --- | --- |
| *1^st^ Round* |  |  |  |
|  | 3795 | Sense | GGCAAGGTCARGAYACTTTAGCCC |
|  | 3851 | Antisense | CTGGTGGGCGTTTAGTTACGCATC |
| *2^nd^ Round* |  |  |  |
|  | 5019 | Sense | CAATATGCTTACTTRACAGTRGGAG |
|  | 5003 | Antisense | TACGCATCYTGGCTGAGGGCACG |

^1^Position in the reference B19V genotype 1 sequence AY386330

TABLE S2

ACCESSION NUMBERS OF (NEAR COMPLETE) B19V GENOME SEQUENCES

*Genotype 1:* AY386330, AJ781031, JN211124, AB126266, JN211162, AJ781035, JN211122, MH201455, AJ781032, KX752821, AJ781038, JN211123, AJ781037, AB126268, KM393165, AB126267, AJ781034, AJ781033, JN211141, JN211181, JN211184, AJ781036, JN211154, JN211166, MH201456, JN211172, JN211174, JN211168, MN746295, JN211135, JN211161, JN211146, JN211126, JN211128, JN211132, MT988400, MT410187, JN211169, MN105993, FN669505, FN669504, MN096574, MN746296, KR005643, JN211152, KM393167, KM393166, KM393168, JN211134, KT310174, JN211173, DQ225148, DQ225149, DQ225150, DQ225151, KC013340, JN211185, JN211140, JN211175, JN211170, KM065415, DQ408301, JN211130, Z70528, KC013329, KC013305, KC013325, KT268312, KC013346, KC013303, KC013327, KC013331, KC013338, KC013333, KC013344, KC013312, KC013314, JN211163, JN211144, JN211183, JN211176, KC013316, JN211155, DQ293995, KC013343, M13178, AF161225, JN211179, JN211129, Z70599, AY504945, MT410189, HQ340602, AB126270, AB030694, AB126265, KM393169, MT410185, MT682520, KM393163, JN211180, KC013308, M24682, MT988402, AF162273, KC013332, AB126269, KC013324, KC013321, Z70560, KM393164, AF161224, AY028237, AB126262, AB126264, AB030673, AB030693, AB126271, FN598217, KY940273, JN211133, JN211148, JN211121, MN096575, MN746294, MN096576, MN737621, JN211165, JN211178, FN669506, JN211137, JN211158, JN211171, JN211142, JN211136, JN211156, FN669503, FN669502, AF161223, MN096573, JN211145, JN211164, JN211125, JN211127, JN211150, JN211151, FN669507, KR005642, KR005644, KR005640, KR005641, JN211157, AF161226, AF113323, JN211138, JN211167, JN211131, JN211159, JN211160, JN211139, JN211182, JN211153, JN211149, JN211177, Z68146, JN211147, JN211143, KM065414, FJ591158, DQ357065, DQ357064, MH151117.

*Genotype 2:* LN680968, DQ333427, HQ340601, MT988398, MT988399, AY064476, AY064475, KF724386, DQ333428, AJ717293, EF216869, AB550331, MT410186, KF724387, MT988397, MT988401, DQ333426, AY903437, AY044266, MT410184.

*Genotype 3:* DQ408303, DQ408304, DQ408302, DQ408305, KC013306, KC013326, AY647977, KC013323, AY582124, DQ234778, KC013310, KC013315, KC013328, DQ234779, AY083234, AY582125, FJ265736, DQ234775, DQ234771, DQ234769, AJ249437.

TABLE S3

FREQUENCY OF POSITIVES ON SERIAL DILUTION OF B19V INTERNATIONAL STANDARD

| **Genotype** | **Primers** | **IU** | **IU/ml** | **Neg** | **N** | **Pos** |
| --- | --- | --- | --- | --- | --- | --- |
| 1 | NS1 | 1 | 25 | 8 | 8 | 0 |
| 1 | NS1 | 2 | 50 | 7 | 8 | 1 |
| 1 | NS1 | 3 | 75 | 4 | 8 | 4 |
| 1 | NS1 | 5 | 125 | 8 | 26 | 18 |
| 1 | NS1 | 5 | 125 | 2 | 8 | 6 |
| 1 | NS1 | 8 | 200 | 2 | 8 | 6 |
| 1 | NS1 | 10 | 250 | 0 | 8 | 8 |
| 1 | NS1 | 50 | 1,250 | 0 | 29 | 29 |
| 1 | NS1 | 500 | 12,500 | 0 | 29 | 29 |
| 1 | NS1 | 5000 | 125,000 | 0 | 13 | 13 |
| 1 | VP2 | 1 | 25 | 5 | 8 | 3 |
| 1 | VP2 | 2 | 50 | 3 | 8 | 5 |
| 1 | VP2 | 3 | 75 | 0 | 8 | 8 |
| 1 | VP2 | 5 | 125 | 4 | 26 | 22 |
| 1 | VP2 | 5 | 125 | 0 | 8 | 8 |
| 1 | VP2 | 8 | 200 | 0 | 8 | 8 |
| 1 | VP2 | 10 | 250 | 0 | 8 | 8 |
| 1 | VP2 | 50 | 1,250 | 0 | 29 | 29 |
| 1 | VP2 | 500 | 12,500 | 0 | 29 | 29 |
| 1 | VP2 | 5000 | 125,000 | 0 | 13 | 13 |
| 2 | NS1 | 1 | 25 | 2 | 8 | 6 |
| 2 | NS1 | 2 | 50 | 0 | 8 | 8 |
| 2 | NS1 | 3 | 75 | 0 | 8 | 8 |
| 2 | NS1 | 5 | 125 | 0 | 29 | 29 |
| 2 | NS1 | 5 | 125 | 0 | 8 | 8 |
| 2 | NS1 | 8 | 200 | 0 | 8 | 8 |
| 2 | NS1 | 10 | 250 | 0 | 8 | 8 |
| 2 | NS1 | 50 | 1,250 | 0 | 29 | 29 |
| 2 | NS1 | 500 | 12,500 | 0 | 27 | 27 |
| 2 | NS1 | 5000 | 125,000 | 0 | 8 | 8 |
| 2 | VP2 | 1 | 25 | 1 | 8 | 7 |
| 2 | VP2 | 2 | 50 | 0 | 8 | 8 |
| 2 | VP2 | 3 | 75 | 0 | 8 | 8 |
| 2 | VP2 | 5 | 125 | 0 | 29 | 29 |
| 2 | VP2 | 5 | 125 | 0 | 8 | 8 |
| 2 | VP2 | 8 | 200 | 0 | 8 | 8 |
| 2 | VP2 | 10 | 250 | 0 | 8 | 8 |
| 2 | VP2 | 50 | 1,250 | 0 | 29 | 29 |
| 2 | VP2 | 500 | 12,500 | 0 | 27 | 27 |
| 2 | VP2 | 5000 | 125,000 | 0 | 8 | 8 |
| 3 | NS1 | 1 | 25 | 5 | 8 | 3 |
| 3 | NS1 | 2 | 50 | 2 | 8 | 6 |
| 3 | NS1 | 3 | 75 | 1 | 8 | 7 |
| 3 | NS1 | 5 | 125 | 1 | 26 | 25 |
| 3 | NS1 | 5 | 125 | 0 | 8 | 8 |
| 3 | NS1 | 8 | 200 | 0 | 8 | 8 |
| 3 | NS1 | 10 | 250 | 0 | 8 | 8 |
| 3 | NS1 | 50 | 1,250 | 0 | 28 | 28 |
| 3 | NS1 | 500 | 12,500 | 0 | 27 | 27 |
| 3 | NS1 | 5000 | 125,000 | 0 | 8 | 8 |
| 3 | VP2 | 1 | 25 | 2 | 8 | 6 |
| 3 | VP2 | 2 | 50 | 0 | 8 | 8 |
| 3 | VP2 | 3 | 75 | 0 | 8 | 8 |
| 3 | VP2 | 5 | 125 | 1 | 26 | 25 |
| 3 | VP2 | 5 | 125 | 0 | 8 | 8 |
| 3 | VP2 | 8 | 200 | 0 | 8 | 8 |
| 3 | VP2 | 10 | 250 | 0 | 8 | 8 |
| 3 | VP2 | 50 | 1,250 | 0 | 28 | 28 |
| 3 | VP2 | 500 | 12,500 | 0 | 27 | 27 |
| 3 | VP2 | 5000 | 125,000 | 0 | 8 | 8 |

TABLE S4

ALL POSITIVE RESULTS (MEAN IU/ML)

| **Sample Name** | **Minipool size** | **NS1 - Mean IU/mL per sample** | **VP2 - Mean IU/mL per sample** |
| --- | --- | --- | --- |
| B110 | 96 | Undetermined | 26.00 |
| B108 | 96 | Undetermined | 50.56 |
| B219/D10 | 1 | Undetermined | 58.46 |
| B219/D2 | 1 | Undetermined | 84.74 |
| B103 | 96 | Undetermined | 84.80 |
| B256/E | 12 | Undetermined | 130.03 |
| B112/C2 | 1 | Undetermined | 386.29 |
| B121 | 96 | Undetermined | 101.59 |
| B303 | 96 | Undetermined | 266.14 |
| B015 | 96 | 2.66 | 70.20 |
| B031 | 96 | 4.32 | 19.03 |
| B291 | 96 | 5.08 | 53.85 |
| B206 | 96 | 5.99 | 55.39 |
| B292 | 96 | 6.34 | 115.87 |
| B212 | 96 | 6.69 | 111.90 |
| B018 | 96 | 10.22 | 38.28 |
| B072 | 96 | 11.60 | 12.81 |
| B061 | 96 | 11.71 | 11.71 |
| B038 | 96 | 18.00 | 40.89 |
| B005 | 96 | 18.77 | 5.05 |
| B202 | 96 | 20.24 | 217.30 |
| B039 | 96 | 20.33 | 22.26 |
| B157 | 96 | 20.77 | 82.81 |
| B112 | 96 | 22.51 | 27.01 |
| B260 | 96 | 22.83 | 38.93 |
| B155 | 96 | 24.24 | 69.11 |
| B083 | 96 | 28.16 | 281.64 |
| B160 | 96 | 28.38 | 144.73 |
| B296 | 96 | 28.80 | 52.86 |
| B231 | 96 | 29.97 | 93.69 |
| B182 | 96 | 31.64 | 98.98 |
| B275 | 96 | 32.97 | 183.02 |
| B159 | 96 | 33.98 | 179.68 |
| B113 | 96 | 34.83 | 33.68 |
| B024 | 96 | 35.93 | 43.63 |
| B079 | 96 | 36.11 | 28.17 |
| B063 | 96 | 36.79 | 49.45 |
| B074 | 96 | 37.90 | 61.02 |
| B176 | 96 | 38.37 | 36.80 |
| B254 | 96 | 41.41 | 62.32 |
| B213 | 96 | 42.05 | 126.00 |
| B274 | 96 | 43.87 | 241.66 |
| B284 | 96 | 45.19 | 52.67 |
| B085 | 96 | 45.80 | 134.03 |
| B222 | 96 | 48.35 | 23.48 |
| B054 | 96 | 49.75 | 60.96 |
| B035 | 96 | 52.93 | 87.19 |
| B228 | 96 | 59.92 | 137.57 |
| B067 | 96 | 62.99 | 119.25 |
| B076 | 96 | 64.85 | 140.78 |
| B188 | 96 | 65.42 | 184.86 |
| B141 | 96 | 67.32 | 61.02 |
| B302 | 96 | 68.10 | 142.08 |
| B008 | 96 | 76.92 | 37.57 |
| B124 | 96 | 77.28 | 123.42 |
| B297 | 96 | 81.55 | 231.56 |
| B235 | 96 | 85.79 | 198.16 |
| B057 | 96 | 86.73 | 197.08 |
| B128 | 96 | 87.78 | 123.83 |
| B092 | 96 | 91.80 | 75.40 |
| B207 | 96 | 99.32 | 237.60 |
| B255 | 96 | 103.33 | 168.82 |
| B230 | 96 | 111.45 | 175.27 |
| B289 | 96 | 116.55 | 381.59 |
| B210 | 96 | 141.50 | 229.16 |
| B245 | 96 | 144.17 | 467.90 |
| B236 | 96 | 144.74 | 283.38 |
| B044 | 96 | 146.15 | 174.04 |
| B192 | 96 | 171.64 | 296.89 |
| B281 | 96 | 177.14 | 423.46 |
| B110/C | 12 | 180.74 | 216.36 |
| B136 | 96 | 184.39 | 77.19 |
| B122/F2 | 1 | 196.31 | 660.63 |
| B179 | 96 | 202.31 | 465.39 |
| B194 | 96 | 210.01 | 255.00 |
| B112/C | 12 | 243.34 | 479.15 |
| B271 | 96 | 247.50 | 823.22 |
| B191 | 96 | 321.75 | 251.16 |
| B190 | 96 | 341.46 | 621.26 |
| B122 | 96 | 342.74 | 490.55 |
| B081/C | 12 | 363.53 | 1960.13 |
| B227 | 96 | 363.58 | 830.18 |
| B081 | 96 | 369.45 | 430.46 |
| B256 | 96 | 521.04 | 1267.72 |
| B242 | 96 | 541.84 | 1028.85 |
| B122/F | 12 | 932.19 | 3001.53 |
| B110/C12 | 1 | 1585.22 | 2788.78 |
| B256/G12 | 1 | 3894.69 | 6265.14 |
| B256/G | 12 | 5346.61 | 12619.52 |
| B081/C3 | 1 | 12534.33 | 29683.36 |
| B280 | 96 | 23698.36 | 16060.39 |
| B122/F11 | 1 | 25247.81 | 29032.10 |
| B280/E | 12 | 43346.75 | 38612.33 |
| B256/G5 | 1 | 63892.10 | 143580.69 |
| B280/E5 | 1 | 615415.48 | 637422.66 |
| B219 | 96 | 1740179.66 | 787726.29 |
| B219/D | 12 | 4519832.03 | 11390106.05 |
| B219/D11 | 1 | 47656300.00 | 131872037.50 |

FIGURE S1

PHYLOGENETIC TREE OF AMPLIFIED SEQUENCES IN THE B19V VP2 REGION

FROM STUDY SAMPLES AND GENOTYPE 1-3 B19V VARIANTS


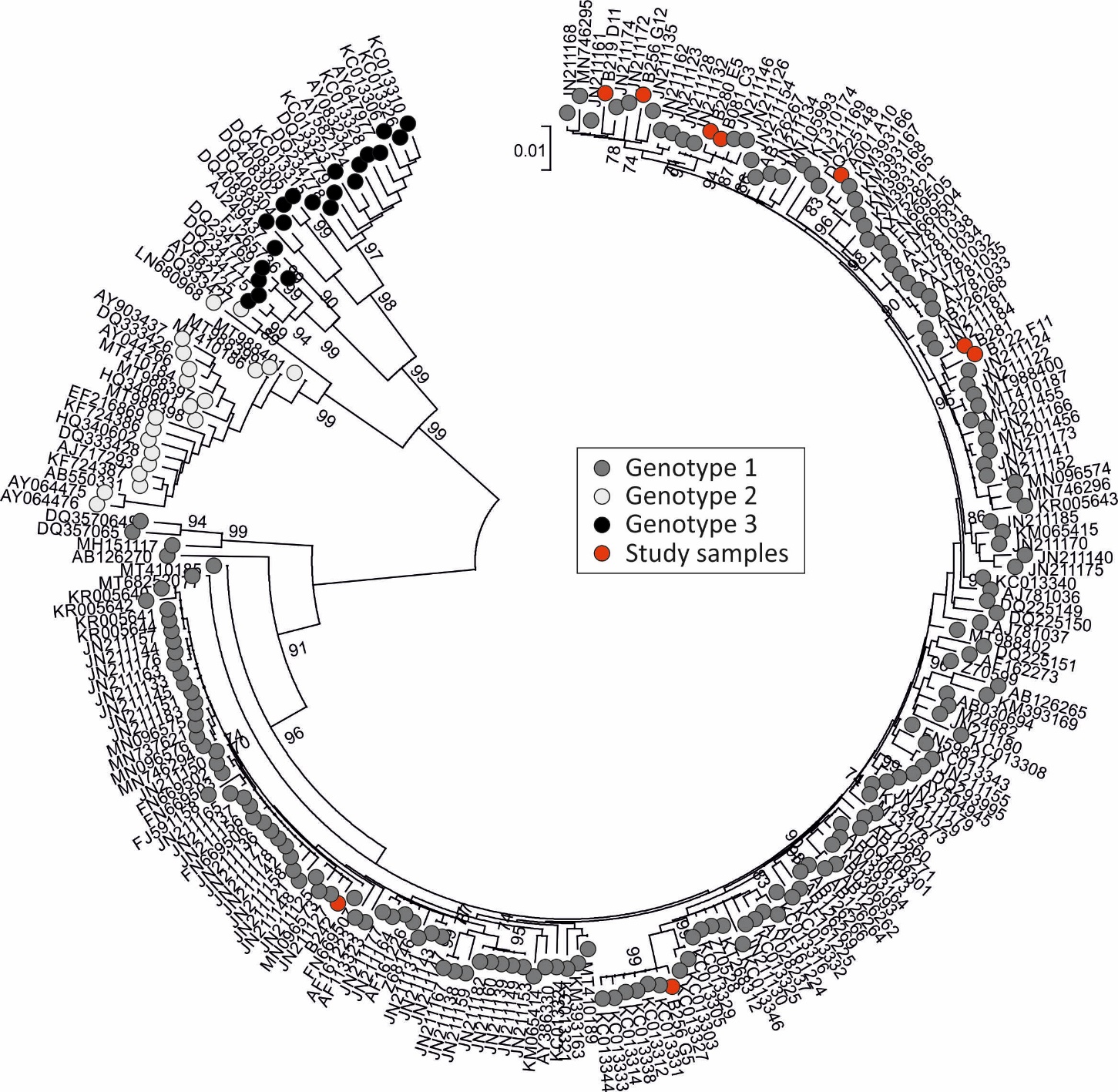


Neighbour-joining tree of VP2 sequences amplified from the study samples and a comparison with published complete genome sequences of B19V genotypes 1-3/ The tree was constructed using maximum composite likelihood distances; the robustness of branches in the tree was estimated by bootstrapping (100 iterations); those supported by 70% of bootstraps are annotated with the bootstrap value.
